# Geographical influences on thyroid abnormalities in adult population from iodine-replete regions: a cross-sectional study

**DOI:** 10.1038/s41598-020-80248-7

**Published:** 2021-01-13

**Authors:** Xiaofeng Wang, Zhe Mo, Guangming Mao, Wenming Zhu, Mingluan Xing, Xueqing Li, Yuanyang Wang, Zhifang Wang, Xiaoming Lou

**Affiliations:** Institute of Endemic Diseases, Department of Environmental Health, Zhejiang Provincial Center for Disease Control and Prevention, 3399 Binsheng Road, Binjiang District, Hangzhou City, 310051 Zhejiang Province People’s Republic of China

**Keywords:** Environmental sciences, Endocrinology, Medical research, Risk factors

## Abstract

The studies on the increasing incidence of thyroid abnormalities are scarce. The aim of this current study was to ascertain the effects of geographical region on thyroid abnormalities under the context of universal salt iodization (USI). We randomly selected 1255 participants residing in inland and 1248 in coast, with the determination of urinary iodine concentration (UIC) and functional and morphological abnormalities of thyroid gland. The median UIC was significantly higher for the inland participants (188.5 μg/L) than the coastal participants (128.5 μg/L; *p* < 0.001), indicating iodine sufficiency in both populations according to the recommended assessment criteria by the World Health Organization. However, the spectrum of thyroid abnormalities varied between regions, with hypothyroidism prevalent in inland and thyroid nodules in coast. The associations between region and thyroid abnormalities via binary logistic regression models showed that the coastal participants were at a higher risk of total thyroid abnormalities than those from the inland (OR 1.216, 95% CI 1.020–1.449), after the adjustment of ten confounders (demographical characteristics, smoking status, metabolism syndrome, and hyperuricemia). These results indicated that further investigations of the adverse effects of hypothyroidism and thyroid nodules on health burden is urgently needed to sustain USI program.

## Introduction

Zhejiang, an eastern province of China, had a history record of severe iodine deficiency disorders (IDD) in 1980s due to low iodine content in the environment (e.g. drinking water, food). Approximately one third of 7–14-year school children were diagnosed with an endemic goiter and more than one hundred of cases with endemic cretinism appeared^1^. In 1995, mandatory universal salt iodization (USI) program was introduced. Subsequently, the secular trend of the prevalence of thyroid abnormalities appears upward with the implementation of USI^2–5^, increased from 22.5–34.3% in 1999–2004^2^ to 46.6–50.9% in 2015–2016^3,5^.

Understanding the causes of thyroid abnormalities plays a critical role on maintaining the implementation of USI for the increasing number of cases with thyroid abnormalities would lose confidence in consumption of iodized salt^6^, which may ultimately result in IDD re-emerging^7^. Previously published studies on the causes of thyroid abnormalities, however, remain inconsistent. Some experts speculated that excessive iodine intake from household kitchen iodized salt contributed to thyroid problems, since the incidence of thyroid abnormalities was significantly increased in a population with more than adequate level of iodine intake when compared with those with optimal intake in regions where iodized salt was a majority dietary iodine source^3,4,8,9^. Some considered low iodine intake as a potential risk factor due to the physiological role of iodine on thyroid gland^10^. There are also some experts who attributed it to the nowadays increased medical detection with sensitive technique devices^11,12^, metabolic syndrome, and personal susceptible factors^13–16^.

The Regulation on Salt Iodization to Eliminate IDD was brought into effect after 1995^17^. All edible salt for both human and animals is required to be iodized in market and non-iodized edible salt is bought via a prescription only at the designated places. Zhejiang is categorized into two parts based on the location of salt factories: the coastal region where salt factories are located in city-level administrative areas; the inland region where there is no salt factories. The coastal region had a long history of the production of edible salt via the evaporation of seawater since it is close to the East China Sea. The residents living in coast were easily access to non-iodized edible salt from these salt factories. Nevertheless, non-iodized salt is unavailable for most of the inhabitants residing in inland^18,19^. It is essential to taking this regional disparity into consideration to understand causes of thyroid abnormalities based on the aforementioned influential factors. Thus, our study focused on the associations between geographical regions (the coast vs. the inland) and the incidence of thyroid abnormalities, after the adjustment of ten confounders (e.g. demographical characteristics, smoking status, metabolic syndrome, and hyperuricemia).

## Results

### Demographical characteristics of the total participants

A total of 2760 adults were invited to participate in this study. Of them, 257 participants were excluded since 9 did not completed the questionnaires, 184 self-reported a history of thyroid abnormalities and 64 did not have blood drawn. The final population for analysis was 2503 participants, with 1255 from the inland and 1248 from the coastal region.

Table 1 showed the characteristics of the total studying participants by region. The median UIC for the participants from the inland (188.5 μg/L) was significantly higher than those from the coast (128.5 μg/L; *p* < 0.001). The coastal participants had a higher educational level and higher income when compared with those from the inland (both *p* < 0.001). The participants from the coast also had a higher percentage of metabolism syndrome (22.8%) than those from the inland (17.6%; *p* < 0.001). No differences in gender, ethnicity, age group, smoking cigarettes, and hyperuricemia were defined between these two regions (all *p* > 0.05).

**Table 1:** Characteristics of the total study participants by region.

Characteristics	Inland (N = 1255)	Coast (N = 1248)	*p*
	%	%	
**Gender**			0.410
Males	50.4	52.1	
Females	49.6	47.9	
**Ethnicity**			0.624
Han group	99.3	99.4	
Non-Han group	0.7	0.6	
**Age group (years old)**			0.133
18.0–29.9	22.9	25.4	
30–39.9	19.3	20.4	
40–49.9	20.2	21.6	
50–59.9	18.2	14.7	
60–69.9	10.8	10.5	
≥ 70	8.6	7.5	
**Educational level (years)**			< 0.001
≤ 9	54.4	55.4	
10–12	23.5	17.1	
≥ 13	22.1	27.5	
**Income per capita (US dollars)**			< 0.001
< 5000	36.2	18.6	
5000–14,999	46.5	65.8	
≥ 15,000	17.4	15.6	
**Median UIC (μg/L)**	188.5 (181.8–195.3)	128.5 (124.0–136.0)	< 0.001
**Metabolism syndrome**	17.6	22.8	0.001
**Hyperuricemia**	11.0	10.3	0.548
**Smoking cigarettes**	44.5	46.2	0.395

### Spectrum of thyroid abnormalities

**Figure 1:**
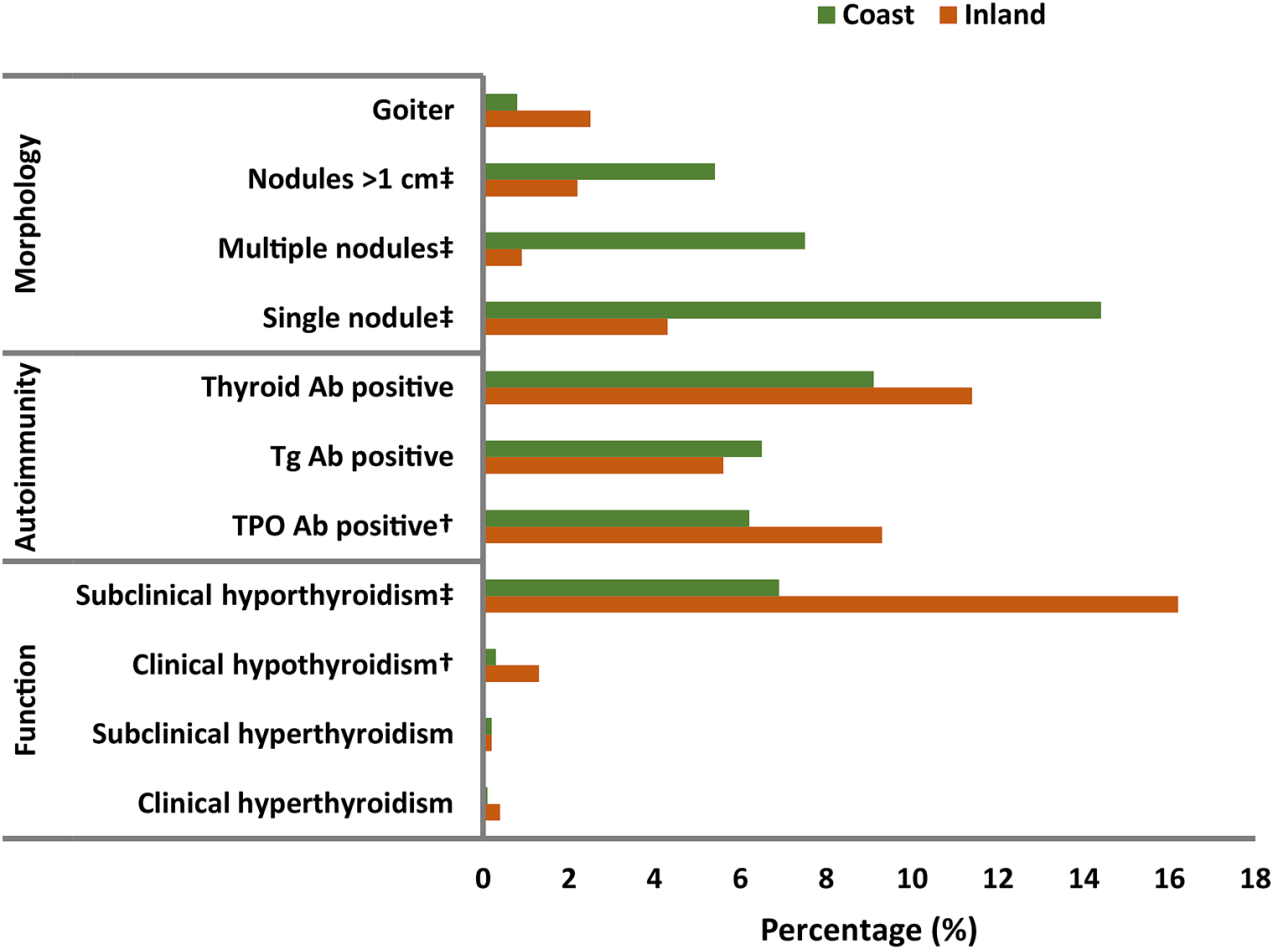
Spectrum of thyroid abnormalities by region. The bar chart showed the difference in incidence of each sort of thyroid abnormalities between the coast and the inland via Chi-square tests (†:*p* < 0.05; ‡: *p* < 0.001). TPO Ab: Thyroid peroxidase antibodies; Tg Ab: Thyroglobulin antibodies. Thyroid Ab: Thyroid peroxidase antibodies or thyroglobulin antibodies. green square box: the coast; orange square box: the inland.

Figure 1 showed the total studying participants with both functional and morphological abnormalities of the thyroid gland were compared between the inland and the coastal regions. In general, hypothyroidism (both subclinical and clinical hypothyroidism) prevailed in the land region whereas thyroid nodules (both single and multiple nodules) dominated in the coast.

The percentage of hypothyroidism was significantly higher for the participants from the inland (17.5%) than those from the coast (7.2%; *p* < 0.001). Nevertheless, the percentage of thyroid nodules was significantly greater for the participants from the coast (22.0%) than those from the inland (5.2%; *p* < 0.001). Both the percentage of subclinical hypothyroidism and the percentage of clinical hypothyroidism were significantly higher for the participants from the inland region than those from the coast, 16.2 vs. 6.9% (*p* < 0.001), and 1.3% vs. 0.3% (*p* = 0.007), respectively. The percentages of participants with single nodule, multiple nodules, or with at least one nodule whose diameter was larger than 1 cm were all significantly higher for those from the coast than from the inland, 14.4% vs. 4.3% (*p* < 0.001), 7.5% vs. 0.9% (*p* < 0.001), and 5.4% vs. 2.2% (*p* < 0.001), respectively. The percentage of TPO Ab positive was significantly higher for those from the inland (9.3%) than those from the coast (6.2%, *p* = 0.003). The goiter rate for those from either the inland (2.5%) or from the coast (0.8%) was lower than the China’s assessment criteria of elimination of IDD (0.5%). No differences in clinical hyperthyroidism (*p* = 0.104), subclinical hyperthyroidism (*p* = 0.996), Tg Ab positive (*p* = 0.338), and thyroid Ab positive (*p* = 0.075) were defined between these two regions. Thus, there was no significant difference in the percentage of total thyroid abnormalities (i.e. total forms of thyroid abnormalities above-mentioned) by region (*p* = 0.130).

### Association between region and thyroid abnormalities

Due to limited cases with clinical hyperthyroidism (n = 6), subclinical hyperthyroidism (n = 4), and goiter (n = 56), we further explored the associations between region and hypothyroidism, thyroid Ab positive, thyroid nodules, and total thyroid abnormalities, before and after the adjustment of the confounders via binary logistic regression models (Table 2).There was no greatly change on odds ratios (ORs) between the unadjusted and other three adjusted models for both hypothyroidism and thyroid nodules. After the adjustment of the confounders in model three, OR for hypothyroidism showed that the participants from the inland was 2.531 times higher risk than those from the coast (95% CI 1.946–3.300; *p* < 0.001). However, there was stronger evidence for adverse effects on thyroid nodules for those in coast when compared with those in inland, with both unadjusted (OR 5.150, 95% CI 3.879–6.837; *p* < 0.001) and the other three adjusted models (OR 5.819, 95% CI 4.337–7.808 in model one, OR 5.820, 95% CI 4.338–7.810 in model two, and OR 5.713, 95% CI 4.253–7.675 in model three; all *p* < 0.001). No significant differences between region and thyroid Ab positive were defined before and after three adjusted models were performed (all *p* > 0.05). Overall, the coastal population were at 1.216 times higher risk for total thyroid abnormalities than the inland population (OR 1.216, 95% CI 1.020–1.449 in model three; *p* < 0.05).

**Table 2:** Risk of thyroid abnormalities in adults, unadjusted and adjusted for potential confounders.

Thyroid abnormalities^a^	Unadjusted model		Adjusted model 1		Adjusted model 2		Adjusted model 3	
	OR (95% CI)	*p*	OR (95% CI)	*p*	OR (95% CI)	*p*	OR (95% CI)	*p*
Hypothyroidism	0.368 (0.284–0.477)	< 0.001	0.392 (0.302–0.510)	< 0.001	0.391 (0.301–0.528)	< 0.001	0.395 (0.303–0.514)	< 0.001
Thyroid Ab positive	0.782 (0.603–1.013)	0.063	0.830 (0.637–1.083)	0.170	0.827 (0.634–1.078)	0.161	0.809 (0.620–1.056)	0.120
Thyroid nodules	5.150 (3.879–6.837)	< 0.001	5.819 (4.337–7.808)	< 0.001	5.820 (4.338–7.810)	< 0.001	5.713 (4.253–7.675)	< 0.001
Total thyroid abnormalities	1.139 (0.962–1.348)	0.131	1.226 (1.030–1.460)	0.022	1.230 (1.033–1.464)	0.020	1.216 (1.020–1.449)	0.029

Model 1: Adjusted for age, gender, education, and income.

Model 2: Adjusted for age, gender, education, income, and smoking cigarettes.

Model 3: Adjusted for age, gender, education, income, smoking cigarettes, metabolic syndrome, and hyperuricemia.

^a^For the independent variable of region, the inland was used as the reference group.

## Discussion

Our study showed that the median UIC for either the inland population (188.5 μg/L) or the coastal population (128.5 μg/L) fell into the range of optimal iodine nutrition according to the WHO’s recommendation^20^, suggesting that adult population in Zhejiang were iodine-sufficient, without regional difference. These figures were consistent with the results of the 2014 China’s national surveillance on IDD^21^. Since more than 90% iodine intake excrete from urine, the disparity in the median UIC between these two regions can be explained by the difference in iodine intake which may be influenced by two factors. First, the coastal population hold widespread misconception that non-iodized salt is one sort of natural salt and more healthy than iodized salt^6^. They are more likely to consume non-iodized salt than those from the inland^6^, as seen from the coverage of household iodized salt, which is usually lower in the coast (61.9–64.3%) when compared with the inland (95.1–95.3%)^22^. Second, a varied dietary intake habit of edible salt was observed between these two regions^23^. The coastal population was accustomed to lighter taste than those from the inland, with 3.4–6.7 g/d of salt intake by weight for a standard male adult in coast vs. 8.9–12.0 g/d in inland^24^, respectively.

This study further showed that the spectrum of thyroid abnormalities varied by region, with hypothyroidism prevailing in inland and thyroid nodules in coast, respectively. It is consistent with the previous studies which showed that iodine prophylaxis in edible salt against IDD changed the pattern of thyroid abnormalities^2,3,25^. However, these results were inconsistent with our previous study, showing that the coastal population had a higher incidence of hypothyroidism than the inland population (11.4% in coast vs. 7.2% in inland), and the inland population had an incidence of thyroid nodules as high as the coastal population (nearly 20%)^26^. The disagreement of these two studies may be a result of different populations selected. The constituent of the participants in our study was based on the census of Zhejiang in 2014, resulting in representative samples of the general adult population. However, the subjects in the latter research included only those with thyroid diseases screened by the examination of thyroid ultrasonic imaging, attributing to an unnegligible selection bias.

Hypothyroidism is more commonly observed in iodine-replete regions. However, it may pose an increased risk of cerebrovascular diseases and coronary heart diseases. Further studies are needed to clarify the distributed characteristics of hypothyroid between these two regions. It is difficult to compare the incidence of thyroid nodules across countries, and even between studies, due to differences in modality of determination, population selection, and variability in iodine supply. Previously observational studies have showed that increased age, female, smoking cigarettes, high BMI, diabetes, inadequate iodine intake and overscreening were independent risk factors for thyroid nodules^27–29^. However, after the adjustment of all potential confounders, the finding in this study was still robust, with a higher incidence of thyroid nodules in the coast than in the inland. In consideration of the same context of long-standing sufficient iodine intake between regions and the same medical detection method performed in this current study, we speculated that there may be no associations between thyroid nodules and inadequate iodine intake, excessive iodine intake, or overscreening. Our study added support that the incidence of thyroid abnormalities varied by region^30^. Whether there is adverse effect of thyroid nodules on health is not well understood. Future researches are needed to study on both the mechanism of the formation of thyroid nodules in iodine-replete regions and the effects on health using larger study cohorts.

Our study has several limitations. First, because this is an epidemiology survey, some confounders that we have not included may contribute to a bias, e.g. genetic variation influencing thyroid hormone action, exposure to different environmental agents. Thus, the findings of this study should not be extrapolated and need to be replicated with large data. Second, each participant with thyroid nodules of which its diameter is greater than 1 cm was not performed a fine-needle aspiration biopsy, but were recommended to thoroughness of the examination.

## Conclusions

This current study confirmed that the spectrum of thyroid abnormalities geographically varied in the iodine-replete regions, with hypothyroidism predominately in inland and thyroid nodules primarily in coast. A comprehensive investigation of the adverse effects of hypothyroidism and thyroid nodules on health burden is urgently needed. We believe that our findings have great public health importance for China to sustain the USI strategy.

## Methods

### Sampling methods

In this cross-sectional study, stratified and multiple-stage random sampling methods were used. First, one county-level administrative division was randomly selected from the inland region (Quzhou, Jinhua, Lishui, and Hangzhou cities) and the coastal region (Wenzhou, Taizhou, Zhoushan, and Ningbo cities), respectively. Second, all towns from each selected county were chosen. Third, at least two communities from each selected town were randomly chosen. Finally, every eligible individual from each community were selected based on the population composition of 2014 census data^31^.

### Participants

The participants were recruited if they had lived in the selected region for more than five years, aged older than 18 years old, and were able to comply with the requirements of the study. The exclusion criteria were those who were pregnant, lactating, taking medications which might alter thyroid function test results (e.g. aspirin, heparin, antiepileptic drugs, steroids, anion exchange resins, or iodine-containing medications)^32,33^, had a history of radiation exposure, acute or terminal illness, used iodinated contrast agents within three months, had a positive history of thyroid abnormalities, and had difficulties to fully communicate. Samples of the morning spot urine and the fasting blood were obtained from each participant between August 2015 and January 2016.

### Laboratory analysis

Urinary iodine concentration (UIC) was determined via the arsenic-cerium catalytic spectrophotometry method based on the Sandell-Kolthoff reaction. For thyroid function tests, the blood serum levels for thyroid-stimulating hormone (TSH), free thyroxine (FT4), free triiodothyronine (FT3), thyroid peroxidase antibodies (TPO Ab), and thyroglobulin antibodies (Tg Ab) were tested by electrochemiluminescence immunoassay method (Roche Diagnostics, Germany). Serum levels of FT4 and FT3 were measured only when TSH level was out of the reference range recommended by the manufactures. For thyroid morphology, B-mode ultrasonography was performed by the trained investigators using a 7.5-MHz linear transducer. Fasting plasma glucose (FPG) was tested with a modified glucose oxidase/peroxidase method (Mindray Biomedical Electronics, Shenzhen, China). Triglycerides (TG), high-density lipoprotein cholesterol (HDL-C), and blood uric acid were determined using the standard enzymatic methods via commercial kits (Mindray Biomedical Electronics, Shenzhen, China).

The coefficient of variation (CV) for the UIC determination method was 1.2%–1.7%, with the recovery of added iodine was 99.2%. The minimum detection limits of TSH, FT4, FT3, TPO Ab, and Tg Ab were 0.014 mIU/L, 0.3 pmol/L, 0.4 pmol/L, 5 IU/mL, and 10 IU/mL, respectively. The intra and inter CV were between 1.1% and 6.3% and between 1.9% and 9.5%, respectively. The sensibility of FPG, TG, HDL-C, and blood uric acid were 0.93 mmol/L, 0.04 mmol/L, 0.07 mmol/L, and 1.97 mmol/L, respectively. Quality control was assessed in a laboratory which participated in the China’s CDC.

### Anthropometric measurements

Waist circumferences (WC) were measured at a medium level between the iliac crest and the lower margin of 12th rib by the trained clinician. Diastolic and systolic blood pressure (BP) readings were taken from the right upper arm in a sitting position with a digital blood pressure monitor (Omron, Kyoto, Japan) after a 10-min rest.

### Biochemical diagnosis criteria

The iodine status assessment criteria recommended by the World Health Organization (WHO)^20^ was adopted. The study population was considered iodine-sufficient when the median UIC reached between 100 and 299 µg/L. Diagnostic criteria for thyroid abnormalities were shown in Table 3. Metabolic syndrome was defined as the status in which elevated waist circumference (≥ 85 cm for females or ≥ 90 cm for males) must be shown based on at least any two of the following traits were displayed: (I) elevated TG: ≥ 1.7 mmol/L, or specific treatment for this lipid abnormality; (II) reduced HDL-C: < 1.04 mmol/L, or specific treatment for this lipid abnormality; (III) elevated BP: systolic B* p* ≥ 130 mm Hg, or diastolic BP ≥ 85 mm Hg, or specific treatment for previously diagnosed hypertension; (IV) elevated FPG: ≥ 6.1 mmol/L, or previously diagnosed type 2 diabetes. Additionally, the cut-off values of uric acid level for hyperuricemia diagnosis were < 420 mmol/L for males or < 350 mmol/L for females. Medical suggestion was provided for any participant with abnormal above-mentioned indicators. Date of birth, region, gender, ethnicity, smoking cigarettes, and a self-reported history of diagnosis of thyroid abnormalities were also recorded.

**Table 3:** Diagnostic criteria for thyroid abnormalities.

Thyroid abnormalities	Diagnostic criteria^a^
Hyperthyroidism	
Clinical hyperthyroidism	TSH < 0.27mIU/L; FT4 > 22.0 pmol/L and/or FT3 > 6.8 pmol/L
Subclinical hyperthyroidism	TSH < 0.27mIU/L; 12.0 pmol/L ≤ FT4 ≤ 22.0 pmol/L and 3.1 pmol/L ≤ FT3 ≤ 6.8 pmol/L
Hypothyroidism	
Clinical hypothyroidism	TSH > 4.2 mIU/L; FT4 < 12.0 pmol/L
Subclinical hypothyroidism	TSH > 4.2 mIU/L; 12.0 pmol/L ≤ FT4 ≤ 22.0 pmol/L
Thyroid Ab positive	TPO Ab > 34.0 IU/mL or Tg Ab > 115.0 IU/mL
TPO Ab positive	TPO Ab > 34.0 IU/mL
Tg Ab positive	Tg Ab > 115.0 IU/mL
Goiter	Thyroid volume > 22.5 mL for females; > 25.4 mL for males
Thyroid nodules	One or more nodules > 5 mm without goiter

*TSH* thyroid-stimulating hormone, *FT4* free thyroxine, *FT3* free triiodothyronine, *TPO Ab* thyroid peroxidase antibodies, *Tg Ab* thyroglobulin antibodies, *Thyroid Ab* thyroid peroxidase antibodies or thyroglobulin antibodies.

^a^The normal reference range for TSH is 0.27–4.2 mIU/L; for FT4, 12.0–22.0 pmol/L; for FT3, 3.1–6.8 pmol/L; for TPO Ab, ≤ 34.0 IU/mL; for Tg Ab, ≤ 115.0 IU/mL; for thyroid volume, ≤ 22.5 mL for females and ≤ 25.4 mL for males.

A written informed consent was provided from each participant. These research protocols were approved by the ethics committee of Zhejiang Provincial Center for Disease Control and Prevention (ZJLLWYH-20140606). All methods were carried out in accordance with the relative guidelines and regulations.

### Statistics analysis

Data were double input in EPI Data 3.1 (Odense, Denmark) and analyzed by IBM SPSS 25 (IBM Corp., Armonk, NY, USA). Figures were drawn via Microsoft Excel 2016. The non-normally distributed variable (UIC) was expressed as median and 95% CI. The normally distributed variable (age) was shown as mean and standard deviation (SD). Regarding to the categorical variables compared between the inland and the coastal regions, gender was categorized into two groups: males and females. Ethnicity also had two groups: Han and non-Han groups. Education was divided into three: (I) ≤ 9 years (middle school and below), (II) 10–12 years (high school), and (III) ≥ 13 years (college and above). Income per capita in 2014 had three groups: (I) < 5000 US dollars (30,000 Chinese Yuan), (II) 5000–14,999 US dollars (30,000–89,999 Chinese Yuan), and (III) ≥ 15,000 US dollars (≥ 90,000 Chinese Yuan). Age was categorized into six: (I) 18.0–29.9 years old; (II) 30–39.9, (III) 40–49.9, (IV) 50–59.9, (V) 60–69.9, and (VI) ≥ 70. Smoking cigarettes was defined as a condition with smoking at least 180 cigarettes during lifetime and every day at the time of enrollment. Categories data were presented as counts and percentages and were compared via Chi-square test between groups.

Binary logistic regression model analyses were used to assess the association between region and thyroid abnormalities, before and after the adjustment of ten confounders including demographical characteristics, smoking status, metabolism syndrome, and hyperuricemia.

## Data Availability

The datasets generated during and/or analysed during the current study are available from the corresponding author on reasonable request.
